# Regional differences and threshold effects of labor transfer affecting the technical efficiency of China’s agricultural industry: A case study of the apple industry

**DOI:** 10.1371/journal.pone.0278348

**Published:** 2023-02-02

**Authors:** Yu Sun, Ruijuan Du, Xinmin Liu, Xiumei Xu

**Affiliations:** College of Economics and Management (Cooperative), Qingdao Agricultural University, Qingdao, China; Universidad Nacional Autonoma de Nicaragua Leon, NICARAGUA

## Abstract

Apples, as a typical agricultural product with high added value, play a significant role in increasing farmers’ income and promoting regional economic growth. They have become one of the main ways for farmers to develop agricultural and sideline products in China’s Loess Plateau and Bohai Rim region. Based on panel data for provinces from 2007 to 2020, this study used stochastic frontier analysis to calculate the technical efficiency of apple production in China’s major apple-producing areas and then introduced urbanization rate as the threshold variable. Based on the quantity, quality, and structure of the rural labor force, the threshold model was used to empirically analyze the effect of labor transfer at different stages of urbanization on industrial technical efficiency in the main apple-producing areas. The results showed that labor transfer had an obvious negative effect on apple production. The labor transfer at the national level has had an obvious negative impact on the output of the apple industry, and the impact of labor transfer on the technical efficiency of China’s apple industry is significantly different; that is, the impact of labor outflow on the technical efficiency of apple production is different in different regions. In some areas, the technical efficiency of production in the main apple-producing areas can be significantly improved. Finally, the proportion of the labor force showed significant differences in its effect on technical efficiency in different stages of urbanization.

## Introduction

As a typical high-value-added industry, apple production has played an important role in China, helping to increase farmers’ income and promote economic growth [1, 2]. Compared with developed countries, however, China’s apple industry is technically inefficient [3, 4]. In addition, as a result of rapid development and the ongoing transfer of the labor force, China’s apple industry faces a number of additional problems [5, 6]. For example, the increase in labor costs associated with labor transfer is especially serious. It is important, therefore, to evaluate the factors affecting the technical efficiency of apple production in China to improve it and reduce regional differences.

To improve the technical efficiency of apple production, it is necessary to optimize the allocation of various production factors such as labor force and land. This requires analyzing the relationship between labor-force transfer and technical efficiency. First, the transfer of the rural labor force can help alleviate the contradiction between humans and land as well as the problem of “overdensity” in agricultural production [7, 8]. Second, many high-quality young and middle-aged rural laborers are migrating to urban areas, causing a decline in the quality of the rural labor force and negatively affecting the apple industry [9, 10]. Third, there are obvious regional differences in economics and natural resources in China, resulting in significant regional differences in the production efficiency of the apple industry.

Regional differentiation in terms of urbanization is increasing in China [10, 11]. According to China’s National Bureau of Statistics, the urbanization rate of the population was 60.60% in 2020, up 1.02 percentage points from the previous year; the highest value was in Shanghai (88.10%) while the lowest was in Tibet [12, 13]. It is clear, therefore, that there are significant economic and environmental differences among the different regions of China. There are also significant differences in the effects of labor transfer on technical efficiency under different degrees of urbanization. It is important, therefore, to examine how to reduce the adverse effects of labor transfer on apple production, improve its technical efficiency, and optimize the allocation of labor resources.

## Literature review

In 2021, the planting area and scale of apple production in China accounted for more than 50% of the global total [14, 15]. However, as a result of labor shifts, the overall quality of apple farmers in China, as well as the gender ratio, has changed dramatically. In recent years, researchers have increasingly investigated this phenomenon. It is generally believed that labor transfer has diverse effects on agriculture and has both advantages and disadvantages for its technical efficiency [14, 16]. Some studies have found that agricultural capitalization promoted by labor transfer can improve the scale of agricultural operations [8, 17]. Moreover, the increase in young and middle-aged laborers engaging in labor transfer can have benefits for economic development [9, 18]. Others, however, have suggested that labor transfer adversely affects the labor supply [10, 19]. Wang et al. [9]and Zhang, Zhang et al. [20] suggested that labor transfer leads to an outflow of high-quality labor, which affects human capital in agriculture. In summary, labor transfer has both advantages and disadvantages. On the one hand, labor transfer from agricultural to nonagricultural areas can improve the overall production efficiency of agriculture. On the other hand, labor force outflow leads to a loss of high-quality labor, which reduces the overall quality of farmers and the overall level of the labor force in agriculture. There remains a need, therefore, to investigate how labor transfer affects the technical efficiency of agriculture.

Studying the effects of changing production modes on apple growers’ economic benefits, Zhang, Shi et al. [14] found significant differences in return on investment between labor-intensive and labor-saving technologies; further, labor-intensive technologies had more significant effects on low-income growers. Using data envelopment analysis (DEA), Jin, Li et al. [15] found that the degree of aging in the agricultural labor force negatively affected apple production efficiency. Su et al. [21], meanwhile, found that although rural labor-force transfer significantly inhibited agricultural production efficiency, the higher the average number of members of farmers’ professional cooperatives, the more they can promote the improvement of agricultural production efficiency and alleviate the negative impact of agricultural labor transfer on agricultural production. Zhang et al. [22] found that labor transfer had a positive effect on farmers’ selection of soil-testing and formula-fertilization technology. Zhang, Yan et al. [23]found that concurrent employment among farmers affected the production efficiency of apple farmers.

Previous studies have used threshold models to analyze China’s rural labor force under different stages of urbanization based on the proportion, quality, and structure of the labor force. However, the effect of labor transfer on the technical efficiency of apple production in China has not been reported. Therefore, this study used stochastic frontier analysis (SFA) to analyze the technical efficiency of China’s apple industry. Specifically, we investigated the effect of labor transfer on regional differences in the technical efficiency of apple production in terms of the structure, quantity, and quality of labor. Finally, by conducting a threshold effect analysis of the urbanization rate index, the differing effects of rural labor transfer in different regions was analyzed.

## Materials and methods

### Research area and data sources

**Fig 1:**
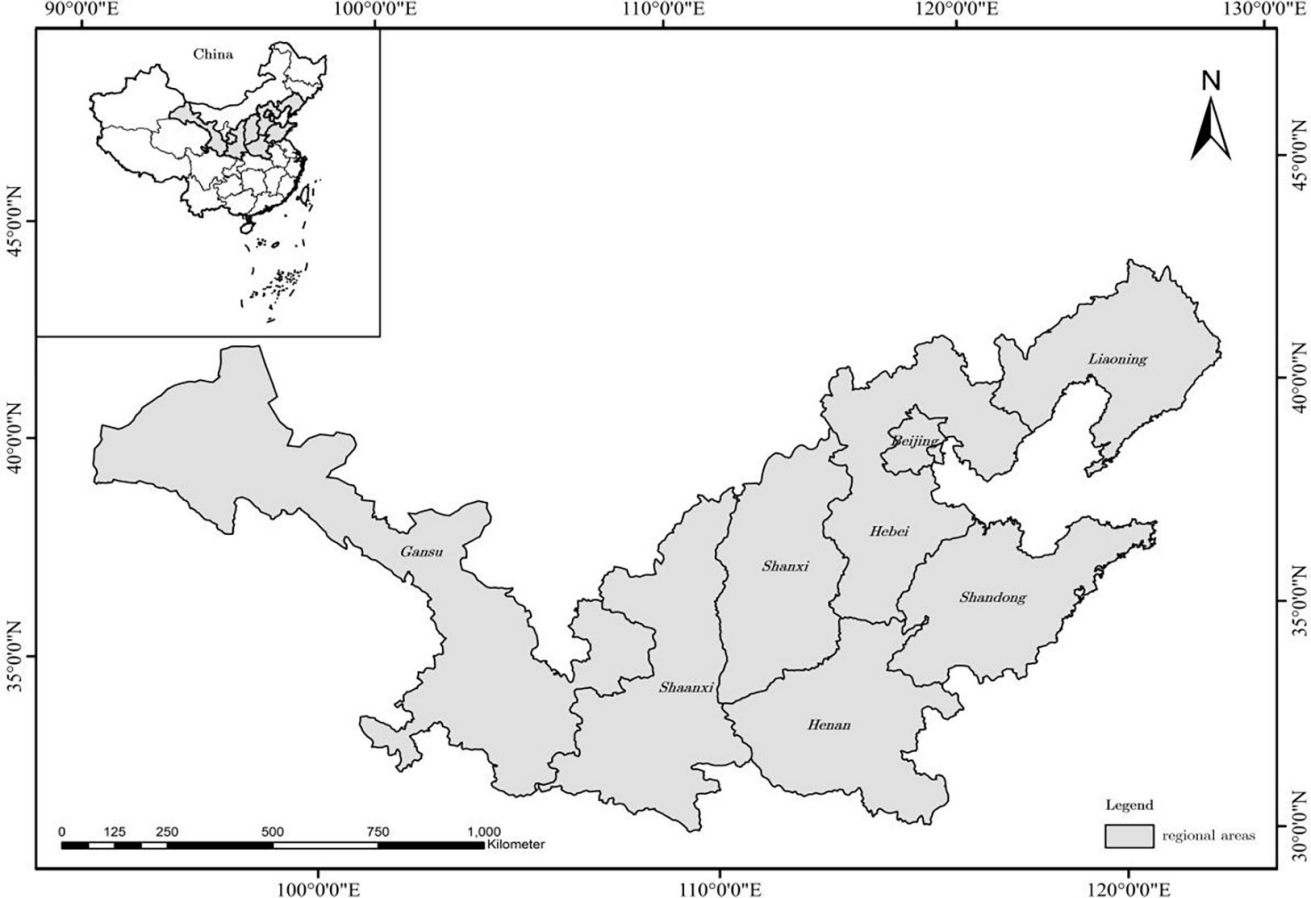
Location of each regional area and its characteristics.

This study investigated eight major apple-producing areas in China from 2007 to 2020. The production and supply capacity of these major apple-producing areas are key to maintaining the security of China’s apple industry [15, 24]. Regional differences among China’s provinces are related to their geographical environments. In 2020, the apple output of eight major apple-producing areas (Beijing, Hebei, Shanxi, Liaoning, Shandong, Henan, Shaanxi, and Gansu) reached 38.803 million tons, accounting for 88.06% of China’s total apple output [25, 26]. In addition, there is more rural labor-force transfer in these main apple-producing areas. Therefore, for research purposes, these areas can be considered representative and well suited for studying the effect of labor-force transfer on technical efficiency. Specifically, we used 2007–2020 panel data for Beijing, Hebei, Shaanxi, Shanxi, Liaoning, Shandong, Henan, and Gansu for the investigation. The data mainly came from the China Statistical Yearbook, China Population and Employment Statistical Yearbook, China Agricultural Product Cost and Income Compilation, China Rural Statistical Yearbook, and the statistical yearbooks of various provinces. The interpolation method was used for certain years with uncollected data. Fig 1 and Table 1 presents the location of all regional areas and their main sociodemographic and productive characteristics.

**Table 1:** Main sociodemographic and productive characteristics of each region.

Region	Main sociodemographic and productive characteristics of each region
*Beijing*	Beijing is the political and economic center area, with sufficient high-quality labor force, and its development focus is high-tech.
*Hebei*	Hebei has a large population, a dense population and a sufficient supply of labor force, which belongs to the apple advantage area in Bohai Bay.
*Shaanxi*	Shaanxi is a big agricultural province, which belongs to the dominant area of Loess Plateau.
*Shanxi*	Shanxi is a big agricultural province and belongs to the dominant area of the Loess Plateau.
*Liaoning*	Liaoning has abundant labor supply and abundant production factors such as land.
*Shandong*	Shandong is a province with a large population and a large agricultural population, with abundant production factors such as labor force and land.
*Henan*	Henan is a populous province with a dense population and sufficient labor supply.
*Gansu*	Gansu and other places, because they live in the interior, are not highly developed, and have rich population supply and natural resources.

### Theoretical framework

We aimed to study the production efficiency of the apple industry after the input of various costs. “Labor-force transfer” is the process in which the main labor force of farmers in rural areas transfers from the agricultural field to nonagricultural fields as a result of increased urbanization; specifically, workers originally engaged in agricultural production flow into cities to find nonagricultural work. We studied labor transfer’s effect on apple production efficiency based on fluctuations in technical efficiency caused by changes in the quality and quantity of the labor force resulting from labor transfer. Since the urbanization rate is the main factor affecting labor-force transfer, the scale of labor-force transfer varies greatly according to different stages of urbanization. Thus, the factors related to labor transfer are mainly reflected in changes in the urbanization rate. We therefore used the urbanization rate as the threshold variable to study the regional differences in and effects of technical efficiency in different urbanization stages.

### Measurement indicators

We initially used SFA to analyze the technical efficiency of China’s main apple-producing areas [27, 28]; one output index and three input indexes were used for measurement. Among them, the index of apple output was the average yield per hectare in each area, and the index of apple production input was the material and service cost, labor cost, and land cost per hectare [29, 30]. Material costs mainly include mechanical operation, fertilizer, pesticide, drainage, and irrigation costs. Labor costs include employment fees and household labor discounts, and land costs mainly include land rent and self-owned land discounts [5, 31].

Data for the quantity, quality, and structure of the labor force were selected as follows: First, labor-force quantity was indirectly replaced by the ratio of the population aged 15–64 to the total population of farmers in the main apple-producing areas. Based on the quantity of the labor force, the ratio of the main labor force in the rural surplus labor force and the speed and scale of labor-force transfer could be clearly seen. The change in the proportion of the main labor force could indirectly reflect the degree and speed of labor transfer. Second, the labor quality index was measured based on farmers’ education level (no school, elementary school, junior high school, high school, technical secondary school, college). We also calculated the proportion of each level of education by introducing the proportion of non-illiterate labor in rural labor in China’s provinces to calculate the degree of education level of the labor force in China’s main apple-producing areas. Third, the labor force structure index was measured by calculating the proportion of women aged 15 and above in the total rural labor force [32, 33]. The core explanatory variable of this study was the technical efficiency of apple production in the investigated areas; the control variables included the irrigation rate and other factors. The proportion of irrigation and drainage fees per hectare in the output value per hectare was replaced by the proportion of irrigation and drainage fees per hectare; the annual urbanization rate of each province in China was taken as the threshold variable. The measured value of technical efficiency in China’s major apple-producing areas was explained through the three main indicators of labor quantity, quality, and structural proportion to measure regional differences in technical efficiency caused by labor transfer [34].

To eliminate differences in each variable dimension, data were normalized using the extremum method. Table 2 shows the descriptive statistics of the variables.

**Table 2:** Descriptive statistics of the variables.

Variable name	Observations (Obs)	Average mean	Standard deviation (SD)	Median	Minimum value	Maximum
*Technical efficiency effect*	126	0.92	0.032	0.920	0.84	0.97
*Total output value*	126	0.493	0.193	0.460	0.000	1.000
*Labor cost*	126	0.279	0.202	0.250	0.000	1.000
*Land cost*	126	0.369	0.254	0.343	0.000	1.000
*Fertilizer fee*	126	0.302	0.215	0.249	0.000	1.000
*Irrigation fee*	126	0.360	0.242	0.316	0.000	1.000
*Pesticide fee*	126	0.390	0.209	0.335	0.000	1.000
*Mechanical fee*	126	0.238	0.174	0.198	0.000	1.000
*Urbanization rate*	126	0.431	0.249	0.389	0.000	1.000
*Proportion of labor force*	126	0.5918	0.185	0.602	0.000	1.000

### Method selection and model construction

#### Measurement of apple’s technical efficiency

At present, methods commonly used to study technical efficiency include parametric SFA and nonparametric DEA [35]. Since this study aimed to investigate the effect of labor transfer on technical efficiency, technical inefficiency needed to be considered. Therefore, SFA was chosen as the main research method [36]. SFA analyzes production processes according to economic principles and uses the results to estimate and design the production function model; then, the obtained parameters are systematically inspected and analyzed [37]. Therefore, SFA can be considered a comprehensive method for analyzing technical efficiency. Referring to Guo Yajun et al. [38], we used SFA to analyze the changing trend of technical efficiency in China’s apple industry. Focusing on the selection of the production function in the model, the following SFA model was established by referring to the literature [39, 40]:

$$\ln Y_{it}=\alpha_{0}+\alpha_{1}\ln A_{it}+\alpha_{2}\ln B_{it}+\alpha_{3}\ln C_{it}+\alpha_{4}\ln D_{it}+\alpha_{5}\ln E_{it}+\alpha_{6}\ln F_{it}+V_{it}-U_{it}.$$

In the model, Y_it_, as the explained variable, represents the yield per hectare of the apple industry in year T in I region; a_0_, a_1_⋯a_6_ represent the parameters to be estimated; A_it_ is the labor cost of the apple industry in year t in the ith region; B_it_ is the land cost of the apple industry in year T in the ith region; and Cit is the fertilizer cost of the apple industry in year T in the ith region. Similarly, D_it_、E_it_, and F_it_ represent, respectively, the drainage and irrigation fee, pesticide fee, and mechanical operation fee per hectare in the ith region in year T. V_it_ is the random disturbance term, which represents the uncontrollable factors of apple production in each area; it refers to the possible effects of some inevitable random factors on apple output. $V_{\mathrm{it}}∼N(0,\delta_{v}^{2})$; U_it_ is the technical inefficiency term, which represents the effect of technical inefficiency on output; $U_{\mathrm{it}}∼N(m_{\mathrm{it}},\delta_{u}^{2})$ and V_it_ are independent of each other.

After obtaining the estimated value of U_it_, the formula ${\mathrm{TE}}_{\mathrm{it}}=e^{-U_{\mathrm{it}}}$ could be used to obtain the technical efficiency value of the apple industry in year T in the ith region. When Uit = 0, it means there was no technical inefficiency in the model, and the apple yield of each area was in a situation of complete efficiency; otherwise, the technical inefficiency term was in the model.

We used the statistic $\gamma =\delta_{U}^{2}/(\delta_{V}^{2}+\delta_{U}^{2})$ to test the applicability of the model. When γ→0, the apple output in all areas was on the production front, which could be solved using the least-squares method. When γ→1, it means there were obvious technical inefficiencies in the model, and it was appropriate to use SFA to solve it.

#### Model for labor transfer’s effect on technical efficiency

Labor transfer is mainly manifested in differences in the proportion of labor quantity, quality, and structure in different regions [17, 41]. To explore the effect of these three indicators on technical efficiency, in addition to the efficiency model described above, an influencing factor model was established as follows:

$$m_{it}=\delta_{0}+\delta_{1}*a_{it}+\delta_{2}*b_{it}+\delta_{3}*c_{it}+u_{it}^{*},$$

where m_it_ is the mean value of the output inefficiency of innovation activities; δ_0_, δ_1_, δ_2_, and δ_3_ are the parameters to be estimated; a_it_, b_it_, and c_it_ are the related indicators of labor transfer; a_it_ is the proportion of labor quantity; b_it_ is the proportion of labor structure; similarly, c_it_ is the proportion of labor quality; and $u_{\mathrm{it}}^{*}$ is the effect of the random disturbance term.

#### Threshold model

Urbanization rate is the main factor affecting labor-force transfer [34, 42]. Based on the technical efficiency analyzed above using SFA, here, we consider the nonlinear relationship between labor transfer and technical efficiency at different stages of urbanization by taking urbanization level as the threshold variable. The panel regression model is expressed as follows:

$$y_{it}=\alpha_{it}+\beta_{it}x_{it}+\in_{it}.$$

The panel data reflect the effects of individuals and time, where α_it_ is a random variable. The technical efficiency of apple production in each area β_it_ is the explanatory variable coefficient. The proportion of the rural labor force in each province was taken as the core explanatory variable. The urbanization rate of each province was introduced as the threshold variable, and ∈_it_ represents the disturbance term.

## Empirical process and results analysis

### Differences in technical efficiency in different regions

**Fig 2:**
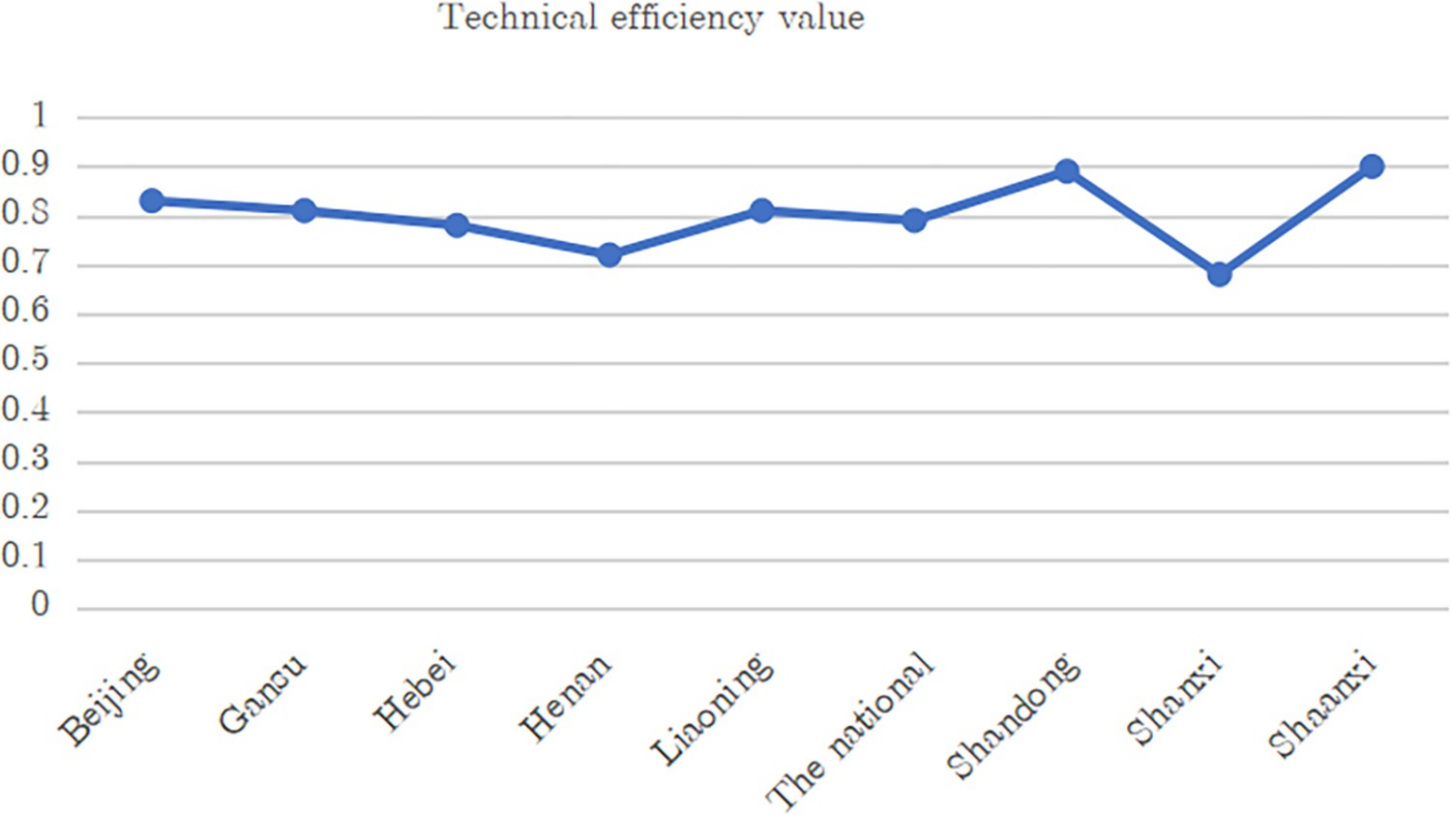
Average technical efficiency of China’s apple industry in different areas.

Based on the model established above, Frontier 4.1 was used to measure the technical efficiency of the apple industry in Beijing, Hebei, Shanxi, Liaoning, Shandong, Henan, Shaanxi, and Gansu, as well as the whole country, during 2007–2020. The influences of the proportion of labor quantity, labor structure, and labor quality on regional efficiency values were analyzed. Frontier 4.1 is software used for SFA research. It can use maximum likelihood to estimate the SFA cost model and production model, among which the BC95 model is the most complex model supported [43, 44]. Based on the efficiency values obtained using Frontier 4.1, Fig 2 presents a broken line chart depicting the average output efficiency of apples in each region during the study period.

The figure shows that technical efficiency at the national level in China was generally higher; the values for most apple-producing areas were between 0.7 and 0.98. As a typical representative of the Loess Plateau area, Shaanxi Province’s technical efficiency value was the highest at 0.98. Shandong Province, as a typical representative of the Bohai Rim region, ranked second at 0.9. This was followed by Beijing, Gansu, Hebei, Liaoning, and other agricultural provinces. Liaoning and Hebei belong to the dominant area of apple production in Bohai Bay while Shaanxi, Shanxi, Henan, and Gansu belong to the dominant area of the Loess Plateau. We found, however, that Beijing, as China’s political and economic center, had the lowest output efficiency among the eight areas. This is mainly because Beijing has developed in the direction of modernization, focusing more on new industries and technology than on agriculture. Meanwhile, Liaoning, Henan, Shandong, and other areas rich in labor and land resources, the efficiency values were generally higher than those of other regions and even the national level. Therefore, owing to ongoing socioeconomic development, labor transfer and various factors of production have affected the technical efficiency of apple production, showing significant regional differences. In the process of urbanization, the rural labor force in some provinces has transferred to urban areas, especially in the rural apple industry, thus affecting its technical efficiency.

### Labor transfer and technical efficiency

Based on the above analysis, differences in technical efficiency generated by input costs in the apple industry could be obtained. On the basis of the technical efficiency values, we introduced the quantity, quality, and structure of the labor force to analyze the effect of labor-force shifts on technical efficiency. Table 3 shows the obtained model estimation results.

**Table 3:** Results for labor transfer’s effect on the technical efficiency of apple production.

Parameter area	Constant term δ_0_	Proportion of labor force δ_1_	Proportion of labor force structure δ_2_	Proportion of labor quality δ_3_	γ	Wald chi-squared value
*Beijing*	0.56**	0.09***	−0.03**	0.59**	0.78	36.89
	(3.05)	(5.15)	(−3.91)	(3.97)		
*Hebei*	0.87***	0.17**	−0.52***	0.16*	0.58	55.13
	(5.12)	(3.21)	(−5.33)	(2.19)		
*Shanxi*	0.98**	0.02*	−0.98**	0.41***	0.71	42.31
	(3.22)	(2.28)	(−3.22)	(6.82)		
*Liaoning*	1.46***	0.11**	−0.22***	0.61	0.82	79.08
	(6.25)	(3.98)	(−6.41)	(1.33)		
*Shandong*	0.68*	0.23*	−0.27**	0.05***	0.67	65.30
	(2.07)	(2.25)	(−3.89)	(5.09)		
*Henan*	−0.91*	0.29***	−0.14**	0.34*	0.75	48.07
	(−2.18)	(6.88)	(−4.08)	(2.31)		
*Shaanxi*	1.84***	0.04***	−1.05*	0.17***	0.60	58.24
	(−5.24)	(5.31)	(−2.04)	(8.22)		
*Gansu*	0.21***	0.10**	−0.99***	0.33*	0.59	34.52
	(6.19)	(4.09)	(−6.31)	(2.85)		
*Nationwide*	0.89***	−0.11	−0.08	−0.09	0.64	62.80
	(12.32)	(1.51)	(1.12)	(0.98)		

Note

***, **, and * indicate significance at the levels of 1%, 5%, and 10% respectively. Values in parentheses are z-test values.

According to the results, the first γ is greater than 0.5 and close to 1, which is significantly not equal to 0, so it is considered that the model selection in this paper is applicable.

First, according to the national parameter measurement results and compared with the t-test value table in econometrics, it can be seen that the impact of the proportion of labor quantity, structure, and quality on the efficiency of apple production technology has not passed the significance test, which is likely to be due to the effect of the three variables in various provinces. Furthermore, the change in the quantity, structure, and quality of surplus labor between different provinces in the process of transfer of rural labor has a counteracting effect on the efficiency of apple production technology. As a result, their impact on the national level is not significant.

Second, according to the results of parameter estimation, it is obvious that there are serious regional differences in the effects of the proportion of labor quantity, structure, and quality on the output efficiency value of the apple industry in various places. (1) Regarding labor quantity, according to the estimated proportion of the labor force in various provinces and cities, it can be seen that in Shandong, Henan, and other provinces where the proportion of young and middle-aged labor is significant, it has also had a positive effect on the efficiency of apple production technology. (2) Regarding the proportion of the labor force structure, it negatively affected apple output efficiency. This indicates that the higher the “feminization” rate, the greater the negative effect on output efficiency. These effects differed by region, showing particularly significant effects in Shanxi, Shaanxi, and Gansu. This further reveals that phenomena such as “feminization” and “aging” brought about by labor transfer in certain regions have contributed to regional differences in the technical efficiency of apple production. (3) Regarding labor quality, the educational level of the rural labor force generally had a negative effect on technical efficiency. The effect of labor force quality was particularly prominent in Beijing. At the same time, labor transfer also leads to a generally low educational level among rural laborers, resulting in significant differences in the effect of labor transfer on agricultural output efficiency in different regions. Notably, the more educated the women are, the more technically efficient the apple farmers are. In apple cultivation, the higher the education level of the female labor force is, the more scientific the production behaviors become, including orchard management, fertilizer application, and pesticide use, and the more likely women are to accept new knowledge and master new technologies.

### Threshold model estimation results

In this study, the factors related to labor transfer were mainly reflected in changes in the urbanization rate. Different levels of urbanization have different effects on the scale of labor transfer. Here, we further introduce urbanization rate as a threshold variable to investigate the effect of labor transfer on technical efficiency in different stages of urbanization.

Stationarity tests of each variable showed that each variable belonged to first-order stationarity. In consideration of possibly serious multicollinearity, we needed to check whether there were serious correlations between variables. Thus, we used 2 VIF (variance inflation factor) to inspect each variable. Values greater than 10 indicate more serious multicollinearity; values below 5 are ideal [45, 46]. Table 4 shows that the average VIF between variables was 2.66—far below 5. Thus, there was no serious multicollinearity between variables.

**Table 4:** Multicollinearity test results.

Variable	VIF value	1/VIF
*Pesticide fee*	4.87	0.205156
*Fertilizer fee*	3.34	0.299505
*Total*	2.75	0.362995
*Mechanical fee*	2.74	0.365106
*Labor cost*	2.30	0.434548
*Land cost*	1.90	0.525204
*Quantity proportion*	1.82	0.550805
*Irrigation fee*	1.57	0.637890
*VIF mean*	2.66	

#### (1) Threshold effect test

Threshold effect refers to the variables in a point mutation phenomenon. With ongoing urbanization, energy efficiency is influenced by many factors. Taking urbanization level as the threshold variable, we used bootstrapping combined with grid search to look for possible mutation points. Table 5 shows the threshold effect results [47, 48].

**Table 5:** Threshold effect test results.

Type	Statistic	p-value	Bootstrapping number	Critical value			Threshold
				10%	5%	1%	
*Single threshold*	17.01	0.0233	300	12.2832	14.7992	20.3616	0.6303
*Double threshold*	5.98	0.5233	300	18.0247	26.9826	46.1444	0.2170, 0.5859,
*Triple threshold*	2.60	0.9167	300	13.7181	16.2216	25.4832	0.1197, 0.2170, 0.5859

**Fig 3:**
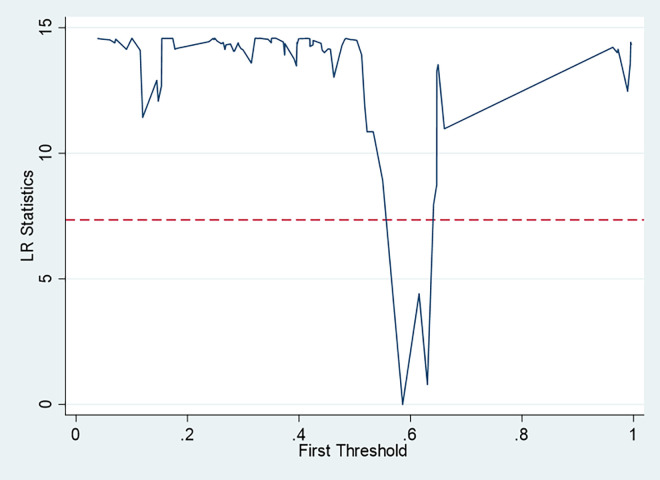
Single-threshold search process.

Table 5 shows that the p-value corresponding to a single threshold was 0.0233. A p-value less than 0.1 is statistically significant. Therefore, a single threshold was applied in this study. The corresponding threshold value was 0.6303, indicating that energy efficiency changes significantly when urbanization reaches 0.6303 (the logarithmic urbanization value). Next, a single-threshold search process needed to be performed, as shown in Fig 3.

#### (2) Model regression results

To further analyze the impact of labor transfer on the technical efficiency of the apple industry, the slope coefficient of each interval was estimated and analyzed based on different threshold value intervals, and the systematic method was used to analyze the threshold effect model. The estimated results are shown in Tables 5 and 6. The regression model was established as follows:

$$y_{it}=\alpha +\beta_{i}x_{it}+\overset{n}{\underset{i=1}{\sum }}\alpha_{i}{\mathrm{Control}}_{it}+\mu_{i}+d_{t}+\varepsilon_{it},$$

where *x*_*it*_ is the independent variable, control represents the control variable, n is the number of control variables, *μ*_*i*_ represents individual effect, *d*_*t*_ represents time effect, and *ε*_*it*_ is the random disturbance term.

$$y_{it}=\alpha +\beta_{1}x_{it}I(q_{it}\leq r)+\beta_{2}x_{it}I(q_{it}>r)+\overset{n}{\underset{i=1}{\sum }}\alpha_{i}{\mathrm{Control}}_{it}+\mu_{i}+d_{t}+\varepsilon_{it},$$

where i = 1, 2, …; n represents different individuals; explained variable *y*_*it*_ represents technical efficiency; explained variable *x*_*it*_ is the amount of labor transfer; threshold variable *q*_*it*_ is the urbanization rate; r represents the threshold value of the model; and I(.) is the index function; the value is 1 when the corresponding conditions are true; otherwise, it is 0. Control represents the control variable, n is the number of control variables, μ_i_ represents individual effect, d_t_ represents time effect, and ε_it_ is the random disturbance term. Tables 6 and 7 show the panel model regression results.

**Table 6:** Regression results for the panel model.

	(1)	(2)	(3)
	Ordinary least-squares ols	Individual time double fixed Fe	Standard error results fe_robust
*Total output value*	0.120	0.228**	0.228*
	(1.15)	(2.38)	(1.99)
*Labor cost*	−0.397***	−0.343***	−0.343
	(−4.92)	(−2.80)	(−1.81)
*Land cost*	−0.170***	−0.101	−0.101
	(−2.63)	(−1.12)	(−0.83)
*Fertilizer fee*	0.141	−0.219*	−0.219
	(1.31)	(−1.83)	(−1.47)
*Irrigation fee*	−0.143**	0.030	0.030
	(−2.25)	(0.31)	(0.20)
*Pesticide fee*	−0.100	0.052	0.052
	(−0.78)	(0.49)	(0.51)
*Mechanical fee*	0.337**	−0.003	−0.003
	(2.62)	(−0.02)	(−0.03)
*Quantity proportion*	0.182**	−0.254**	−0.254**
	(2.31)	(−2.16)	(−2.36)
*intercept_cons*	0.816***	1.050***	1.050***
	(13.37)	(11.96)	(11.43)
*N*	108	108	108
*r2*	0.389	0.691	0.691
*r2_a*	0.339	0.586	0.624
*Root-mean-square error (RMSE)*	0.127	0.083	0.079

Note

*** p<0.01

** p<0.05

* p<0.1.

**Table 7:** Regression results for the panel threshold model.

	(1)	(2)	(3)
	Individual fixed	Individual time double fixed	Robust results
*Labor force ratio 1*	−0.193**	−0.208*	−0.208*
	(−2.28)	(−1.83)	(−2.14)
*Labor force ratio 2*	−0.478***	−0.481***	−0.481**
	(−4.33)	(−3.57)	(−2.78)
*Total output value*	0.148*	0.167*	0.167
	(1.92)	(1.79)	(1.53)
*Labor cost*	−0.466***	−0.360***	−0.360*
	(−5.78)	(−3.09)	(−2.07)
*Land cost*	−0.167**	−0.181**	−0.181
	(−2.09)	(−2.01)	(−1.47)
*Fertilizer fee*	−0.136	−0.188	−0.188
	(−1.20)	(−1.65)	(−1.39)
*Irrigation fee*	0.030	0.034	0.034
	(0.36)	(0.37)	(0.28)
*Pesticide fee*	−0.006	−0.036	−0.036
	(−0.06)	(−0.34)	(−0.40)
*Mechanical fee*	0.080	0.046	0.046
	(0.78)	(0.45)	(0.51)
*intercept_cons*	1.094***	1.102***	1.102***
	(16.11)	(12.92)	(10.98)
*N*	108	108	108
*r2*	0.662	0.723	0.723
*r2_a*	0.598	0.625	0.659
*Root-mean-square error (RMSE)*	0.081	0.079	0.075
*F*	19.598	10.311	

Note: t-values in brackets

*** p<0.01

** p<0.05

* p<0.1.

In Table 6, Model 1 is the OLS regression results, Model 2 is the dual fixed-panel individual time model, and Model 3 is the robust standard error results of Model 2. Based on the results in Tables 6, 7 presents a further analysis of the regression results for the panel threshold model.

In Table 7, Model 1 is the regression result of the individual fixed-panel threshold, Model 2 is the result of the individual time double fixed-panel threshold, and Model 3 is the robust standard error results of Model 2. As can be seen in Table 7, with urbanization reaching different stages, the growth and change in the proportion of labor force in the core explanatory variable have a significant negative effect on technical efficiency.

Based on the above analysis, there was a single threshold in the model, and the p-value was 0.0233, which is statistically significant. The corresponding threshold value was 0.6303, indicating that technical efficiency changes significantly when urbanization reaches 0.6303 (which is the logarithmic urbanization value).

The proportion of labor force 1 represents the effect of the proportion of the labor force on technical efficiency when the urbanization rate is lower than the threshold value of 0.6303. When urbanization is below 0.6303, each unit increase in the labor-force proportion will cause an average decrease of 0.208 units in technical efficiency. This shows that when the urbanization level is not high, much of the population is concentrated in agriculture, resulting in a large surplus rural labor force, which in turn intensifies laziness among workers and thus is not conducive to improving the technical efficiency of apple production. Thus, labor transfer caused by urbanization can positively affect rural areas, mitigate the contradiction between people and land, and enhance technical efficiency.

The proportion of labor force 2 represents the effect of labor force proportion on technical efficiency when the urbanization rate is higher than the threshold value of 0.6303. However, when urbanization is above 0.6303, an increase of one percentage point in the labor force proportion will reduce technical efficiency by 0.481 units on average. With high-level urbanization, there is more labor outflow, and the loss of a skilled workforce is not conducive to enhancing rural labor force quality. Moreover, a large amount of waste emerges in rural areas, which is not conducive to improving technical efficiency.

## Discussion and policy implications

### Discussion

The rural labor force in China is increasingly abandoning farms and going to the cities for work. These agricultural migrant workers are mostly young, well educated, and male. Thus, the effective labor force engaged in agricultural production has shown a downward trend. Against this background, this study took the apple industry as an example to study regional differences in and the threshold effect of labor transfer on the technical efficiency of the industry. A threshold model has not been previously used in the literature to empirically analyze the effect of labor transfer under different stages of urbanization on the technical efficiency of apple production in China. Our results confirmed that changes in the quantity, structure, and quality of surplus labor in different provinces can offset the effect of technical efficiency on apple production. This is consistent with Min et al. [49]. and Bradfield et al. [50]. Moreover, there were prominent regional differences in the effect on the output efficiency of the apple industry. It can be seen that the labor force remains one of the most important input factors in apple production. After labor-force transfer, the efficiency of resource allocation is reduced, and newly obtained resources are not effectively invested in apple production, thus affecting overall production efficiency [51, 52]. Therefore, when promoting rural labor-force transfer, attention should be paid to ensuring the adjustment and allocation of factors in apple and other labor-intensive industries to improve efficiency and output on the basis of stabilizing the existing productivity.

The results showed that the effect of labor transfer on apple production efficiency varied significantly in different regions. First, in terms of the proportion of labor force in different regions, labor force, land, and other production factors in Shandong and Liaoning are richer than those in other regions, which indirectly caused technical efficiency in those areas to be significantly better than that in other regions. Because Gansu and some of the other areas are located inland, development levels are not high, leading to an outflow of high-quality labor and technical efficiency values lower than those of Shandong and other areas. Meanwhile, as China’s political and economic center, Beijing has attracted a large amount of high-quality labor, but its technical efficiency in apple production is low because its development focus is mainly on high tech. As a result, land and other production factors are scarce. Second, regarding labor quality, educational level had negative effects on technical efficiency. Labor transfer tends to result in high-quality talent leaving agriculture, which means the educational level of the surplus agricultural labor force is generally low. This is not conducive to improving technical efficiency. Moreover, regarding the labor force structure, labor transfer (mostly by men) results in a higher proportion of females in rural areas and an increase in their share of labor. This is also not conducive to improving technical efficiency. Different from the male labor force, rural female laborers generally still need to take care of their family responsibilities, which puts them at a disadvantage with regard to labor.

Factors causing regional differences in the technical efficiency of apple production are as follows. First, under different urbanization levels, the changes in provincial labor transfer quantity have a significant negative impact on apple production efficiency. When the urbanization level is low, rural labor outflow can relieve the contradiction between humans and land, increasing apple production technical efficiency. However, when the urbanization level is high, the loss of a high-quality labor force can also have a significant negative impact on apple production efficiency. This finding is supported by existing research results—that is, the advancement of urbanization process drives the transfer of surplus agricultural labor to non-agricultural industries, which is conducive to the improvement of the scale and intensification of agricultural production, and optimizes the industrial structure and resource allocation efficiency of the apple industry [33]. Second, regional characteristics have a significant positive impact on apple production efficiency. This finding is consistent with previous studies on the impact of information transfer on farmers’ uptake of innovative crop technologies [8]. For example, in Shandong Province, apple is the main crop supporting local economy. The Shandong government actively provides various policy guarantee and technical support to farmers. In addition, farmers who receive a wide range of knowledge and professional technology training related to apple production show high levels of initiative in learning. Therefore, the joint efforts of the government and farmers can further increase the production efficiency of local apples.

In summary, various problems associated with labor transfer in China have greatly affected the technical efficiency of the apple industry, resulting in significant regional differences.

### Policy implications

First, the moderate “return” of labor force should be promoted. Increasing the effective supply of labor is the basic factor promoting the transfer of labor-intensive industries from coastal areas to inland and border areas. Both the central and local governments should do more to improve the labor market system and promote market-oriented labor mobility. In particular, we need to appropriately increase investments in education, medical care, social security, infrastructure, and public services in less-developed areas to create a developed environment that can attract labor to return.

Second, the matching effect of labor skills and industrial technology level should be paid attention to. Talent bonuses and R&D innovation are important factors promoting the high-end development of the agricultural industry. Although this is a consensus of economics, the maximum value of the two can only be realized under the condition of appropriate labor allocation efficiency. With the continuous popularization of education and the continuous improvement of education level in China, there is an underutilization of labor skills and productivity due to the failure of labor skills to match the industrial technology structure. Therefore, on the basis of further increasing R&D investment in the apple industry and promoting the continuous improvement of industrial technology, it is necessary to pay attention to the matching of labor skills and industrial technology, improve the allocation mechanism of labor skills, and realize the transformation and upgrading of the apple industry as soon as possible.

Third, China should strengthen investment in infrastructure in rural areas so that labor transfer can adapt to the pace of urbanization, and the two can develop in a coordinated way. Rural disadvantages should be addressed to narrow the gap between urban and rural areas, promote overall living standards in rural areas, reduce the gap between rich and poor, and reduce the loss of highly skilled workers through labor transfer, all of which will help improve technical efficiency in apple production. We should also reduce regional differences in technical efficiency. First, we should give full play to the regional advantages of different provinces, relying on their superior land and other production factors, and strive to further develop the characteristics of the apple industry. Second, there should be more preferential policies for the apple industry in rural areas, including preferential investment and subsidies.

Fourth, the level of human capital should be improved in rural areas. The government should enhance the scale of educational investment in rural areas to fundamentally enhance rural human capital. First, the government should continue to increase investment in rural education and introduce relevant policies and plans to increase the high school and college enrollment rates in rural areas on the basis of universal compulsory junior middle school education to promote the education level of the rural labor force (especially the female labor force). Second, the government should strengthen the rural labor force by introducing advanced production technology and management methods, improve the overall education level in rural areas, fully eliminate the labor transfer to the disadvantages brought by the parts, promote apple production technologies, carry out education technology training, and strengthen the cultivation and extension education of apple production technology.

## Conclusions and limitations

### Conclusion

1. Our results showed that from 2007 to 2020, the technical efficiency of China’s main apple-producing regions and that of the whole country were generally high (see Fig 1). Shandong, which has a large population, had the highest apple output (0.89) among the eight regions while Gansu and Beijing had the lowest. This finding is supported by previous research results—namely, production efficiency is generally higher in the dominant apple-producing areas.
2. Under different levels of urbanization, changes in the amount of labor transfer have had significant negative effects on apple production efficiency. When the level of urbanization is low, labor transfer can alleviate the contradiction between people and land in rural areas and improve the technical efficiency of apple production to a certain extent. However, when urbanization develops to a certain level, the loss of high-quality labor will also have a negative effect on technical efficiency.
3. In different stages of urbanization, the growth of and change in the proportion of the labor force had significant negative effects on technical efficiency. There was a single threshold in the model, and the threshold value was 0.6303, indicating that significant changes will occur when the urbanization rate reaches this value. This suggests that with lower urbanization levels, labor transfer caused a large number of workers to move from agriculture to nonagricultural fields. This can help to relieve the contradiction between people and land and also improve technical efficiency in apple production and other fields.

### Limitations

Certainly, due to the lack of research conditions in various aspects and the restricted perspective of researchers, there are still many shortcomings in this paper. In addition, the research on the impact of labor employment can be further developed and improved in the following aspects: (1) Owing to the limitations of data availability, the historical period of the research is short, and there are few actual research data. (2) This study did not consider the degree of part-time employment of farmers—that is, the effect on production efficiency of farmers who work in cities while engaged in apple production. This will be further discussed and analyzed in future research. (3) Special research can also be carried out on the aged labor force, female labor force, and lowly educated labor force engaged in the apple planting industry, especially in terms of in-depth research on the employment principle, motivation, and psychology of special labor force groups using relevant theories and methods.

## Supporting information

S1 Data (XLSX)
